# Characterization of Plastic Scintillator Detector for In Vivo Dosimetry in Gynecologic Brachytherapy

**DOI:** 10.3390/jpm14030321

**Published:** 2024-03-20

**Authors:** Antonio Herreros, José Pérez-Calatayud, Facundo Ballester, Rosa Abellana, Joana Neves, Joana Melo, Luis Moutinho, Jordi Tarrats-Rosell, Sergi Serrano-Rueda, Luca Tagliaferri, Elisa Placidi, Angeles Rovirosa

**Affiliations:** 1 Fonaments Clínics Department, University of Barcelona, 08036 Barcelona, Spain; rabellana@ub.edu (R.A.); or rovirosa@clinic.cat (A.R.); 2 Radiation Oncology Department, Hospital Clínic Universitari, 08036 Barcelona, Spain; tarrats@clinic.cat (J.T.-R.);; 3 Radiation Oncology Department, Hospital Universitari i Politècnic La Fe, 46026 Valencia, Spain; perez_jos@gva.es; 4 Radiation Oncology Department, Hospital Clinica Benidorm, 3501 Alicante, Spain; 5 IRIMED, IIS-La Fe-Universitat de Valencia (UV), 46100 Burjassot, Spain; facundo.ballester@uv.es; 6 NU-RISE S.A., PCI—Creative Science Park, 3830-352 Ilhavo, Portugal; info@nu-rise.pt (J.N.); joanasmelo@nu-rise.pt (J.M.); moutinho@nu-rise.pt (L.M.); 7 Dipartimento di Diagnostica per Immagini e Radioterapia Oncologica, Fondazione Policlinico Universitario A. Gemelli IRCCS, 00168 Rome, Italy; luca.tagliaferri@policlinicogemelli.it (L.T.); elisa.placidi@policlinicogemelli.it (E.P.); 8 Gynecologic Cancer Unit, Hospital Clínic Universitari, 08036 Barcelona, Spain

**Keywords:** brachytherapy, in vivo dosimetry, plastic scintillator dosimeter

## Abstract

(1) Background: High dose gradients and manual steps in brachytherapy treatment procedures can lead to dose errors which make the use of in vivo dosimetry (IVD) highly recommended for verifying brachytherapy treatments. A new procedure was presented to obtain a calibration factor which allows fast and robust calibration of plastic scintillation detector (PSD) probes for the geometry of a compact phantom using Monte Carlo simulations. Additionally, characterization of PSD energy, angular, and temperature dependences was performed. (2) Methods: PENELOPE/PenEasy code was used to obtain the calibration factor. To characterize the energy dependence of the PSD, the signal was measured at different radial and transversal distances. The sensitivity to the angular position was characterized in axial and azimuthal planes. (3) Results: The calibration factor obtained allows for an absorbed dose to water determination in full scatter conditions from ionization measured in a mini polymethylmethacrylate (PMMA) phantom. The energy dependence of the PSD along the radial distances obtained was (2.3 ± 2.1)% (*k* = 1). The azimuthal angular dependence measured was (2.6 ± 3.4)% (*k* = 1). The PSD response decreased by (0.19 ± 0.02)%/°C with increasing detector probe temperature. (4) Conclusions: The energy, angular, and temperature dependence of a PSD is compatible with IVD.

## 1. Introduction

The high dose gradients and the different manual steps in brachytherapy (also known as interventional radiotherapy) treatment procedures can lead to dosimetry errors, making the use of in vivo dosimetry (IVD) highly recommended for verifying brachytherapy treatments. Some of the causes of deviations between the planned doses and IVD are associated with anatomical variations in patients and technical brachytherapy equipment discrepancies (wrong source calibration, incorrect applicator reference distances, swapped needle connections). Other causes of deviations correspond to uncertainties in IVD [1]. Some detector uncertainties, such as repeatability, signal-to-noise ratio, linearity, and precision of dwell time measurements in patient treatments, were reported in relation to a plastic scintillator detector (PSD) for brachytherapy IVD in a previous study [2]. There are also other important issues, such as detector calibration and its energy, temperature, and angular dependence, that need to be characterized for use in routine clinical practice.

Calibration of an IVD should be performed by cross-comparison with some conveniently calibrated detector for the type of source used [3,4]. One option would be indirect calibration of the absorbed dose in water from the air kerma strength value obtained by a calibrated well chamber or by an in-air-calibrated Farmer ionization chamber [5]. Once the reference air kerma rate (RAKR) is obtained according to the AAPM Task Group n° 43 (TG43) methodology [6], using the consensus data [7], the absorbed dose rate in water can be obtained. 

Another more direct option with less uncertainty would be intercomparison of the detector with an ionization chamber calibrated directly in an absorbed dose to water. However, this method has two important drawbacks. First, there are no accredited laboratories that offer absorbed dose to water calibration of the usual ionization chambers in the quality of Ir-192, the most widespread radioisotope worldwide in remote afterloading systems. Second, although the scattering conditions of the different factors of the AAPM TG43 correspond to a water sphere with a radius of 40 cm, the use of a large water scanning phantom for this purpose in routine clinical practice is not always practical, for example if a check of the calibration factor just before each brachytherapy procedure is needed. To solve both difficulties, a small polymethylmethacrylate (PMMA) phantom was designed and manufactured to calibrate an in vivo detector quickly and robustly in high dose rate (HDR) brachytherapy with a Farmer ionization chamber. To this end, it was necessary to obtain the corresponding factors in AAPM TG-43 using Monte Carlo simulations and to convert the ionization, produced by an Ir-192 source measured in the designed PMMA mini-phantom geometries with a cobalt-calibrated Farmer ionization chamber, into water-absorbed dose values in full scatter geometry.

The present study is innovative because a quality factor for Ir-192 HDR brachytherapy in non-reference AAPM TG43 conditions was obtained in the geometry of a compact PMMA cylindrical phantom (10 cm × 10 cm). 

Polystyrene and an organic fluor made of long aromatic hydrocarbons are the constituents of a PSD. At megavoltage photon energies, these molecules absorb ionizing radiation in a similar way to water. For this reason, these types of scintillators are commonly considered to be tissue-equivalent ionizing radiation detectors in high-energy spectra [8]. However, this assumption must be verified in a spatial varying spectrum, such as that of Ir-192, which can lead to variations in detector response relative to water, defined as the energy dependence of the detector. To test the energy dependence of a PSD, an away-along dose rate table measured with the detector was acquired and compared to the gold-standard absorbed dose rate table from AAPM report 229 [7]. The present study includes this comparison, which to the best of our knowledge has not yet been published for a PSD. 

The variation in the response of a detector with the radiation incidence angle is known as the angular dependence of the dosimeter. Scintillation detectors exhibit some angular dependence due to their length–diameter ratio and manufacturing imperfections, such as optical fiber–scintillator gluing. Increasing the length of the scintillator increases the active volume and, consequently, the signal detected, but it penalizes the angular dependence. Thus, a compromise for the length of the scintillator must be found between the collected signal and the angular dependence of the PSD [9]. In a detector with a cylindrical symmetry, such as the PSD of the present study, two principal planes were defined for specifying the angular dependence—the axial plane, which cuts the cylindrical scintillator perpendicular to its axis, and the azimuthal plane, which contains the axis of the cylindrical scintillator. PSDs are assumed to have low axial and azimuthal angular dependence if the ratio of length to diameter is below 5:1 [9] in comparison with other types of detectors, such as inorganic scintillators [10,11], MOSFET [12], or diodes [13]. However, the high dispersion of values for PSDs reported in the bibliography both in the axial plane (range 0.3–5%) [14,15,16,17] and the azimuthal plane (range 0.6–97%) [9,15,16,18] suggests that precise characterization of this angular dependence is recommended for clinical dosimetry.

Despite the low temperature dependence of PSDs [9], it is known that the PSD signal in the case of BCF-12 decreases linearly with increasing temperature [15,16,17,19,20,21,22]. All these studies used kilovoltage, megavoltage, or Co-60 sources of radiation, but BCF-12 PSD temperature dependence has not yet been reported under Ir-192 HDR source irradiations. From the work of Wootton et al. [20] we know that the generation of Cerenkov light is temperature independent. However, optical coupling (gluing) between the PSD and the optical fiber has some temperature dependence that should be verified in each new detector. In the present study, we investigated the temperature dependence of a BCF-12 PSD coupled to a 1.5 m optical fiber probe with Ir-192 HDR irradiation. 

The purpose of this study was to obtain a fast and robust calibration procedure of PSD probes for brachytherapy IVD, and correction factors for the angular, energy, and temperature dependences of the detector.

## 2. Materials and Methods

### 2.1. Equipment

The equipment used in this work was the prototype PRO-DOSE version 2020 (NU-RISE, Ilhavo, Portugal) IVD system that incorporates an organic scintillator, BCF-12 (Saint Gobain Crystals, Paris, France), with a length of 2 mm attached to a 1.5 m long PMMA optical fiber with a core diameter of 0.5 mm. The core of the PSD is synthesized with polystyrene and fluorescent dopants, and the cladding is made of PMMA. Signal processing and analysis was described in a previous work [2]. The phantom measurements were carried out in a 10 cm × 10 cm cylindric PMMA phantom. The phantom has 4 holes located in the phantom periphery for Ir-192 source insertion using needles with an internal diameter of 1.22 mm at 4 cm from the phantom axis and one central accessory to insert the PSD probe or the PMMA Farmer ionization chamber model 30013 (PTW, Freiburg, Germany).

### 2.2. Monte Carlo Simulations and Experimental Validation

**Figure 1:**
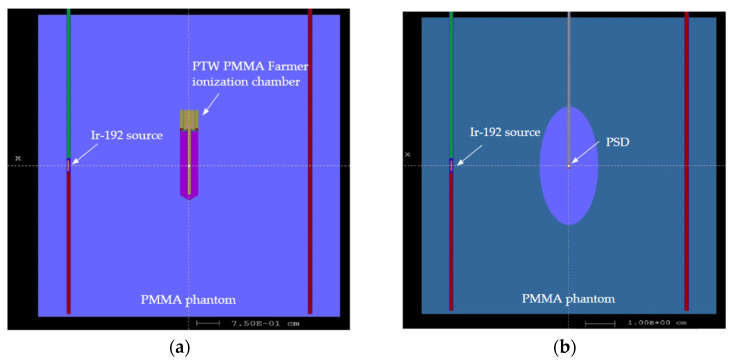
Set-up of the Monte Carlo simulation in the mini PMMA phantom with the Ir-192 source model on the left needle. (**a**) Set-up with the PTW PMMA Farmer ionization chamber. (**b**) Set-up with the PSD in the central axis. The ellipsoid used to eliminate the secondary electrons generated outside its volume is also shown.

The computer code used for the Monte Carlo simulations is the PENELOPE/PenEasy [23]. Absorbed dose to water, polystyrene, and air (i.e., D_w_, D_pol_, and D_air_, respectively) were computed using an Ir-192 source model [24] in two geometries: the 10 cm × 10 cm cylindrical PMMA phantom and a water sphere with a radius of 40 cm [6,25] (full scatter conditions) to obtain the k_QQ0_ quality factor through International Atomic Energy Agency Technical Report Series (IAEA TRS)-398 formalism [26]. The obtained factor allows for absorbed dose to water determination in full scatter conditions from the ionization measured in the PMMA phantom. D_air_ simulations were performed with a model [27] of the PTW PMMA Farmer ionization chamber from the data provided by the manufacturer, averaging the absorbed dose in the air cavity (Figure 1a). To obtain D_air_, a dry air density of 1.205 mg/cm^3^ was used for the standard temperature of 20 °C and an atmospheric pressure of 101.325 kPa, as recommended in Supplement 2 of the 2004 update of the AAPM TG No. 43 Report [6]. The composition as a percentage of dry air mass recommended is 75.527% N, 23.178% O, 1.283% Ar, and 0.012% C, although it differs slightly from what is recommended in report International Commission on Radiation Units and Measurements (ICRU) 90 [28]. D_w_ values were obtained by substitution of the Farmer chamber by using a small water volume as the detector material, with a volume with the same geometry as the PSD (Figure 1b). The density of degassed liquid water used was 0.998 g/cm^3^ at 20 °C. There is no difference in density compared to the corresponding 22 °C value [7]. The Ir-192 spectrum was obtained from the National Nuclear Data Center [29]. The variance reduction technique used was “interaction forcing” (IF = 200), which allows for a considerable reduction in the calculation time. This technique generates forced interactions along the particle path. To compensate for this excess probability of interaction, the average free path is proportionally reduced, and an appropriate statistical weight is assigned to any secondary particle or energy loss resulting from those interactions. 

The cut-off energy was 1 keV for electrons in the detector volume and 10 keV in the rest of the phantom. For the transport of photons, the cut-off energy was 1 keV in all volumes. To optimize the simulations, an additional 8.4 cm^3^ ellipsoid external to the detector was generated. The size of this ellipsoid was selected so that the secondary electrons generated outside the volume did not reach the detector. Only particles of that volume were used for the transport of radiation in the detector (Table 1).

Experimental validations of the Monte Carlo D_air_ ratios obtained were performed with a PMMA ionization chamber in the PMMA phantom and under saturated scattering conditions in an RFA 300 water scanning phantom model (Scanditronix, Uppsala, Sweden) with a positioning accessory replicating the geometry of the mentioned PMMA phantom. For a radial distance of 4 cm from the radioactive source, the scattering differences between a water scanning phantom and the 40 cm radius sphere are negligible [25]. Stainless steel metal needles (AISI 316) 5F (ELEKTA, Veenendaal, The Netherlands) with an internal diameter of (1.22 ± 0.03) mm were used in the PMMA phantom. For the measurements in the water scanning phantom, 4F ProGuide needles with an internal diameter of 1.1 mm were used. The Farmer ionization chamber, connected to a PTW UNIDOS E electrometer, was used for the ionization measurements from irradiations performed with the Ir-192 microSelectron HDR v2 source installed in afterloader microSelectron v3 Digital (ELEKTA, Veenendaal, The Netherlands).

Additional absorbed dose Monte Carlo simulations were conducted in a polystyrene cylinder with the same dimensions as the PSD of the present work (0.5 mm diameter by 2 mm length) in order to compare the experimental measurements in both types of phantoms.

### 2.3. Away-Along Table

The spatial sensitivity of the detector around the brachytherapy Ir-192 source is conditioned by the energy dependence of the PSD, because the spectrum varies at different distances from the source and at varying angular positions relative to source axis due to anisotropy. To characterize this, the signal of the detector was measured at different radial and transversal distances from the effective point of measurement of the PSD to the center of the Ir-192 source using a PMMA phantom submerged in a water scanning phantom. The PMMA phantom has two identical circular plates with holes drilled each centimeter from its central axis to a distance of r = 6 cm. The drilled holes allow the vertical insertion of 294 mm plastic 4F Proguide needles (ELEKTA, Veenendaal, The Netherlands) to project the Ir-192 source at different vertical positions ranging between z = −7 cm and z = +7 cm.

Although metallic needles are more robust than plastic catheters, they have two major drawbacks. First, they are supplied with shorter lengths, which limits the level of water above the needle to achieve full scatter. Second, they are not optimal to characterize the away-along table, because the attenuation produced depends on the obliquity of the irradiation, and its theoretical correction in the Ir-192 spectrum is complicated. 

An additional plastic needle parallel to the afterloader connected needles was positioned in the axis of the phantom to insert the PSD probe or a dummy probe (a probe without a scintillator). The Ir-192 source reference distance and its central dwell position were calculated to coincide with the height of the effective point of the scintillator, defined as z = 0. The dummy probe measurements are needed to correct the signal from the stem effect, caused by the emission of Cherenkov radiation and the fluorescence light generated in the PMMA optical fiber, because irradiations at different radial and vertical distances generate distinct stem effect signals. 

### 2.4. Angular Dependence

The sensitivity of the detector to the angular position of a brachytherapy source at 6 cm from the center of the PSD was characterized in the axial and azimuthal planes measuring the signal of the detector as a function of the angle from 30° to 330° in 20° steps in the axial and the azimuthal planes using a PMMA phantom submerged in a water scanning phantom. The PMMA phantom has two identical circular plates with holes drilled at 6 cm from its central axis every 20° to vertically insert 294 mm plastic 4F Proguide needles (ELEKTA, Veenendaal, The Netherlands) to project the Ir-192 source. For the axial dependence measurements, another plastic needle parallel to the peripherical needles was positioned in the axis of the phantom to insert the PSD probe. For the azimuthal dependence measurements, an additional disc plate was drilled to insert a horizontal needle for the PSD probe or a dummy probe. The needles inserted in each of the channels are perpendicular to this plate. The reason for repeating the measurements in the azimuthal set-up using a dummy probe is to correct the signal from the stem effect, because irradiations at different angles generate distinct stem signals, unlike in the axial set-up. The Ir-192 source reference distance and its dwell position were calculated to coincide with the height of the effective point of the scintillator in both cases.

### 2.5. Temperature Dependence

All the irradiations to characterize the temperature dependence of the PSD were carried out at a controlled room temperature of (25 ± 1) °C. During the complete interval of irradiations (2 h) the RAKR of the source varied less than 0.1%. The measurements were performed by irradiating the scintillator and probe at a practically constant dose rate while varying the temperature in the range from 15 °C to 40 °C and registering the signal of the PSD with the associated software of the PRO-DOSE equipment. 

To control the heating of the water, a hotplate (model LKTC-B1-T; Vevor, Rancho Cucamonga, CA, USA) with a temperature sensor was used. This hotplate includes a magnetic stirring device to homogenize the temperature of the water. Water temperature was measured with two calibrated thermometers (reader: model HD 2107.1, sensor: TP472 I.O, Delta OHM, Milan, Italy) at two heights in the beaker: one (T_sup_) with a sensor 1 cm above the effective point of the PSD and another (T_inf_) at 1 cm below to obtain the most accurate assessment of the PSD temperature. The thermometers were calibrated in a secondary standard laboratory in the range 15–40 °C, in 5 °C steps, and the maximum deviation obtained was 0.04 °C, with an expanded uncertainty (*k* = 2) of 0.09 °C. 

Sets of five irradiations were carried out for each temperature at 5 °C steps within the interval [15 °C, 40 °C]. First, the hotplate setting was adjusted to bring the water to the desired temperature. Before each irradiation set, the water temperature was kept constant for an additional 10 min after stabilization, enabling the PSD to reach thermal equilibrium with the water. After that, the PSD probe was irradiated. The temperature was measured with both thermometers immediately before and after each set of five irradiations. The values were averaged to obtain $T_{initial}$ and $T_{final}$, respectively. Finally, for each set of irradiations the temperature was considered as the mean of $T_{initial}$ and $T_{final}$. 

Assuming a linear variation with temperature in the PSD signal, *S(T)*, the measured values can be adjusted to a simple linear equation, as follows:

(1) $$\frac{S(T)}{S(T_{0})}=1+a\cdot (T-T_{0})$$

where $T_{0}$ corresponds to a temperature of 25 °C (the usual room temperature), and *S*_0_ is the mean signal obtained at this temperature value.

The estimated slope “a” and its uncertainty can be expressed as follows: ($a\;\pm \;u_{T}$)%/°C, where *u_T_* is the combined total uncertainty of Type A and Type B uncertainties (*k* = 1). 

### 2.6. Uncertainties

We derived Type A uncertainties from the standard deviation of a set of measurements. Each measurement was repeated at least three times. Type B uncertainties include electrometer uncertainties stated in a device’s calibration certificate. In specific tests, such as the study of the temperature, we accounted for additional Type B uncertainties, including the uncertainty in the temperature measurement. The uncertainty in the temperature measurements was calculated according to international recommendations [30] as the quadratic sum of Type A and Type B uncertainties. Type B uncertainty includes (a) the thermometer calibration uncertainty and (b) the fact that temperature was measured only before and after irradiations: $T_{initial}$ and $T_{final}$, respectively. Assuming a uniform probability density function, the uncertainty is the interval amplitude divided by $\sqrt{12}$:

(2) $$u_{T}=\frac{T_{initial}-T_{final}}{\sqrt{12}}$$

#### 2.6.1. Monte Carlo Uncertainties

In all simulations, a sufficient number of histories were used to obtain a statistical uncertainty value in the detector material of below 1%. Table 2 summarizes the estimated uncertainties of the Monte Carlo simulations.

#### 2.6.2. Experimental Uncertainties

Experimental uncertainties of the Farmer chamber ionization measurements have been detailed in Table 3.

## 3. Results

### 3.1. Monte Carlo Simulations and Experimental Validation

IAEA TRS 398 formalism [26] gives the following expression for the factor $k_{Q,Q_{0}}$:

(3) $$k_{Q,Q_{0}}=\frac{{\left(s_{w,air}\right)}_{Q}\cdot {\left(W_{air}\right)}_{Q}\cdot p_{Q}}{{\left(s_{w,air}\right)}_{Q_{0}}\cdot {\left(W_{air}\right)}_{Q_{0}}\cdot p_{Q_{0}}}$$

The mean energy expended in air per ion pair formed $\left(W_{air}\right)$ can be considered similar in both qualities [28] (p. 40). Assuming that the Bragg–Gray principle is valid, the absorbed dose in water $\left(D_{w,Q_{0}}\right)$ is related to the mean absorbed dose in the air of the chamber cavity $(D_{\;air,Q_{0}}^{mean}$):

(4) $$D_{w,Q_{0}}=D_{\;\;\;\;\;\;\;\;\;air,Q_{0}}^{mean}\cdot {\left(s_{w,air}\right)}_{Q_{0}}\cdot p_{Q_{0}}$$

And the same holds for any other quality *Q*:

(5) $$D_{w,Q\;}=D_{\;\;\;\;\;\;\;\;\;air,Q}^{mean}\cdot {\left(s_{w,air}\right)}_{Q}\cdot p_{Q}$$

Substituting both equations in Equation (3) yields the following:

(6) $$k_{Q,Q_{0}}=\frac{D_{w,Q}/D_{\;\;\;\;\;\;\;\;\;air,Q}^{mean}}{D_{w,Q_{0}}/D_{\;\;\;\;\;\;\;\;\;air,Q_{0}}^{mean}}$$

Following IAEA TRS 398 formalism [26], the absorbed dose to water in the PMMA phantom using an Ir-192 source can be obtained from the following Equation (7):

(7) $$D_{w,Ir-192}^{\;\;\;\;\;\;\;\;\;\;\;\;\;\;\;\;PMMA\;phantom}=N_{D,w,Co-60}\cdot M^{PMMA\;phantom}\cdot k_{Ir-192}^{\;\;\;\;\;\;\;\;\;\;\;\;\;\;PMMMA\;phantom}$$

where $N_{D,w,Co-60}$ is the Farmer chamber Co-60 calibration factor, $M^{PMMA\;phantom}$ is the temperature and pressure corrected ionization measured in the PMMA phantom, and the $k_{Ir-192}^{\;PMMMA\;phantom}$ factor can be estimated by the Monte Carlo (Equation (6)) from the following:

(8) $$k_{Ir-192}^{\;\;\;\;\;\;\;\;\;\;\;\;PMMA\;phantom}=\frac{{\left(D_{w}/D_{\;\;\;\;\;\;\;\;\;air}^{mean}\right)}_{Ir-192}^{PMMA\;phantom}}{{\left(D_{w}/D_{\;\;\;\;\;\;\;\;\;air}^{mean}\right)}_{Co-60}^{IAEA\;TRS\;398\;geometry}}$$

To compare IVD measurements with treatment planning system (TPS)-calculated values following AAPM TG-43, the quantity of interest is not $D_{w,Ir-192}^{\;PMMA\;phantom}$, but $D_{w,Ir-192}^{\;full\;scatter}$, because we want to measure ionization in the PMMA mini-phantom, but we want the absorbed dose in the full scatter geometry of AAPM TG-43. Using a new factor *F*:

(9) $$D_{w,Ir-192}^{\;\;\;\;\;\;\;\;\;\;\;\;\;\;\;\;full\;scatter}=N_{D,w,Co-60}\cdot M^{PMMA\;phantom}\cdot F$$

*F* can be solved from Equations (7)–(9), as follows:

(10) $$F=\frac{{\left(D_{w}^{\;\;\;\;full\;scatter}/D_{\;\;\;\;\;\;\;\;\;air}^{mean\;\;\;\;\;\;PMMA\;phantom}\right)}_{Ir-192}}{{\left(D_{w}/D_{\;\;\;\;\;\;\;\;\;air}^{mean}\right)}_{Co-60}^{\;\;\;\;\;\;\;\;\;\;\;\;\;\;IAEA\;TRS\;398\;geometry}}$$

In this work, the denominator ratio ${\left(D_{w}/D_{air}\right)}_{Co-60}^{\;IAEA\;TRS\;398\;geometry}$ has not been simulated; it was obtained directly from IAEA TRS 398 [26] for the PTW PMMA Farmer ionization chamber, as follows: ${\left[s_{w,air}\right]}_{Q_{0}}\cdot p_{Q_{0}}=1.112$. 

The Monte Carlo obtained factor was *F* = 1.103 ± 2.5% (*k* = 1). PENELOPE/PenEasy physics (Clinic Monte Carlo) dominates the Monte Carlo component uncertainty, while the contribution of the source positioning inside the needles is the highest uncertainty in the experimental measurements with the PTW Farmer chamber, as indicated in Table 2 and Table 3.

As shown in Table 4, the assumed statement that polystyrene can be considered as water equivalent (in the sense that its absorption and scattering properties are similar) is an approximation with a difference of around 2% in Ir-192 spectra at 4 cm for both ratios.

The discrepancies between the measured ionization ratio and Monte Carlo $D_{air}$ ratios from Table 4 in both geometries are within their experimental and Monte Carlo uncertainties. Monte Carlo uncertainties include material composition, mass–energy absorption coefficients, and tally statistics. The Farmer measurement uncertainties considered are phantom hole drilling accuracy, source positioning, measurement reproducibility, and $N_{D,w,Co-60}$ calibration by the standard laboratory.

### 3.2. Away-Along Table

**Figure 2:**
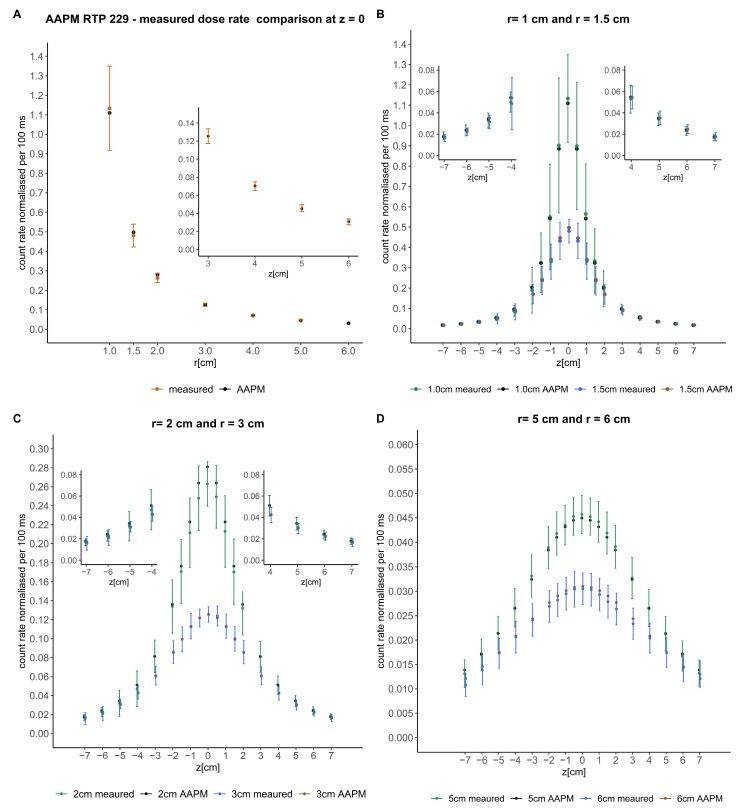
Comparison of the signals detected with the consensus away-along absorbed dose rate table of the microSelectron HDR v2 source of the AAPM report 229 [7]. The crosses represent the mentioned published data and the circles the measured data. Error bars represent the uncertainty. Magnifications of some parts of the comparisons are included to better visualize the data. (**A**) shows the comparison in the perpendicular axis of the source (z = 0). (**B**–**D**) show the comparison at different radial distances from the source in the z-axis.

After processing the detected signals, including subtraction of the stem effect and RAKR correction, the values were compared with the consensus away-along absorbed dose rate table of the microSelectron HDR v2 source of AAPM report 229 and plotted in Figure 2. The PSD count rate detected was normalized to the value of the AAPM report at 4 cm. The steep gradient of the absorbed dose rate near the source is associated with a higher uncertainty in the signal of the detector. The error bars include the uncertainty in (a) longitudinal source dwell positioning, (b) lateral displacement of the source inside the 4F catheter, and (c) noise-to-signal variation with distance. The latter is the dominating contribution to uncertainty at larger distances, while the longitudinal positioning of the source is the most relevant factor close to the source. The measured values obtained deviate from the expected absorbed dose rate by (2.7 ± 2.5)% (*k* = 1) across the published absorbed dose rate table [7]. In the z-axis profiles, the maximum percentage difference corresponded to short radial distances and was below 6.5%. Along the radial distances, in the z-origin (z = 0), the mean and standard deviation of the percentage difference was (2.3 ± 2.1)% (*k* = 1).

### 3.3. Angular Dependence

**Figure 3:**
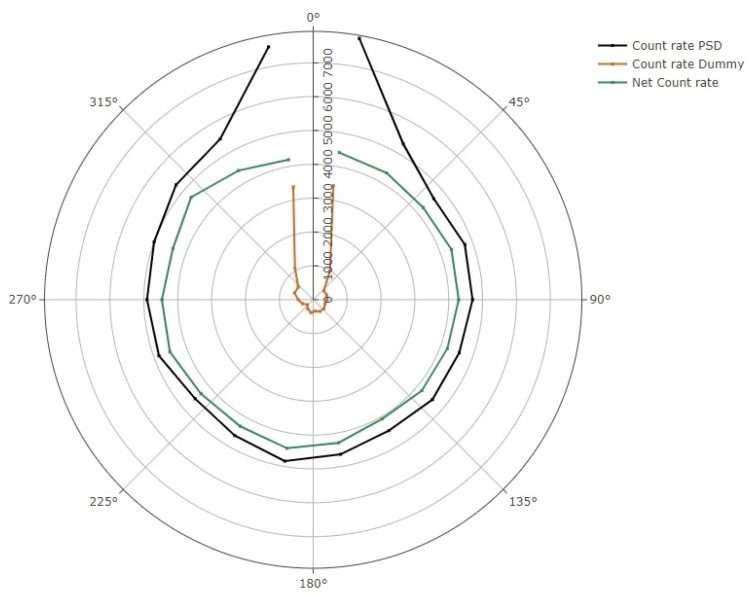
Radar diagram showing the azimuthal angular dependence of the detector. PSD and dummy probe signal are expressed in count rate per 100 ms. The subtraction of the stem effect is also shown.

A radar diagram of the azimuthal angular dependence is shown in Figure 3. It is evident that at angles near the PSD or dummy probes, the stem effect is considerable. To remove this contribution, the subtraction of both signals has also been represented. Taking this correction into account, the mean percent response variation for angles from 30° up to 330° was (2.6 ± 3.4)% (*k* = 1). Signal acquisitions were repeated three times for each angle. The RAKR of the Ir-192 source (38.3 mGym^2^h^−1^) varied less than 0.2% during the measurements, and, thus, no decay correction was applied to the signals obtained.

The response of the dosimeter in the axial plane for angles from 30° up to 330° was (1.9 ± 2.4)% (*k* = 1). The RAKR of the Ir-192 source corresponding to the measurements in the axial set-up was 42.0 mGym^2^h^−1^. The source could be positioned to an accuracy of 1.0 mm. An uncertainty of 1.0 mm in source positioning would result in less than a 0.1% change in signal at the distance of 60 mm used in the axial angular dependence study.

### 3.4. Temperature Dependence

**Figure 4:**
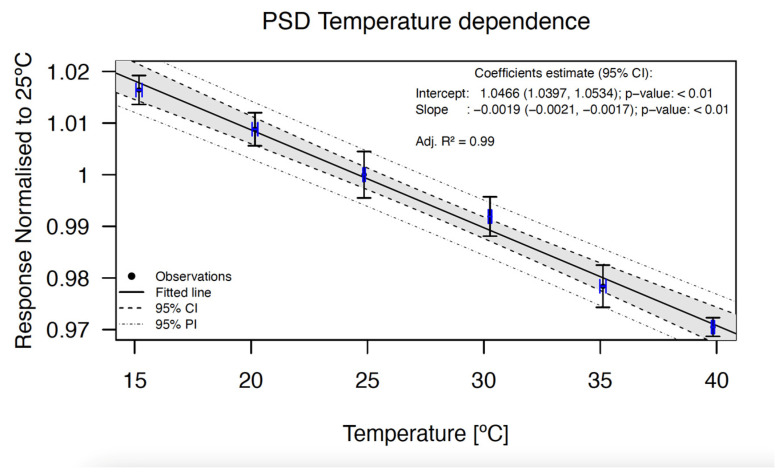
Temperature dependence of the BCF-12 PSD. The points represent the response of the detector normalized to 25 °C. The data show an excellent linear behavior. The correlation coefficient, the intercept, and the slope of the fit are shown at the top right in the graph. The error bars in both axes indicate the uncertainty with a coverage factor of *k* = 1.

The PSD response decreased with increasing detector probe temperature, as shown in Figure 4. A (0.19 ± 0.02)%/°C (a = −0.0019 ± 0.0002) decrease was observed when the water temperature was increased from 15 °C to 40 °C. 

The detector temperature response shows a decrease of (−2.28 ± 0.24)% between measurements at room temperature (25 °C) and body temperature (37 °C). 

## 4. Discussion

### 4.1. Monte Carlo Simulations and Experimental Validation

Instead of using an indirect calibration using air kerma strength, $S_{k}$, to determine the absorbed dose to water, $D_{w}$, transference through AAPM TG43 formalism, a faster, more robust, and direct $D_{w}$ determination procedure was developed for PSD calibration. This is a new method, which to the best of our knowledge has not yet been described previously for in vivo detector calibration. The main advantage of this small PMMA phantom procedure is that PSD calibration can be performed without using the water scanning phantom, optimizing time resources. The full procedure takes less than 10 min and can be performed before each brachytherapy treatment. 

### 4.2. Away-Along Table

Based on the away-along table comparison, the use of the PSD for IVD requires low energy-dependent correction factors. The obtained differences (2.3 ± 2.1)% (*k* = 1) in this study are much lower than the deviations reported with an inorganic scintillator detector by another group [34] in Ir-192, which obtained deviations of (5.2 ± 4.7)% (*k* = 1). The percentage differences (8.6%) obtained with microMOSFET detectors by Ruiz-Arrebola et al. [12] are also much higher than the values obtained with PSD in this work. The energy-dependence corrections needed for a ZnSe:O crystal reported by Jørgensen et al. [35], in which the energy dependence changed by 50% from 20 to 40 mm, are also much higher than the values obtained in the present study. These results corroborate the idea that the energy dependence behavior of the PSD is compatible with IVD for HDR brachytherapy. 

### 4.3. Angular Dependence

A smaller angular dependence was expected for measurements obtained by irradiations around the PSD in the axial plane rather than in the azimuthal plane, due to the cylindrical geometry of the scintillator. This hypothesis was demonstrated with the values measured in both set-ups. The small axial angular dependence is related to the assembly of the PSD, because it is a complex task to achieve perfect alignment of the scintillator and the optical fiber in the gluing process. The angular dependence of the PSD probe in the azimuthal plane showed an increasing signal when irradiated at angles near the optical fiber probe (10° and 350° angles, and to a lesser extent 30° and 330° angles) due to a higher stem effect. On the other hand, the signal of the PSD was similar for irradiations coming from the distal tip of the scintillator (170° and 190° angles) to measurements at angles from 50° to 310°. 

The azimuthal angular dependence of the PSD could be reduced by shortening the length of the scintillator at the expense of a reduction in the signal. The characterization with an Ir-192 source of the azimuthal angular dependence of the BCF-12 scintillator used in this study showed values very similar to the results obtained by other authors [15,18] considering the measurement uncertainties. The axial angular dependence obtained with our detector was also similar to the recent values reported by another group [14]. This is the first time that angular dependence deviations were reported for both axial and azimuthal set-ups with Ir-192 source irradiations. 

### 4.4. Temperature Dependence

This study shows that the PSD shows a temperature dependence of (−0.19 ± 0.02)%/°C, making the application of a correcting factor necessary for IVD. PSDs are usually calibrated at room temperature (25 °C) and then used for IVD at 37 °C. The detector will have a signal (2.28 ± 0.24)% lower than the planned one if not corrected for temperature dependence. As stated by Wootton et al., with measurements with pairs of PSDs, the temperature dependence for PSDs built with similar scintillating fibers is nearly identical [20]. Therefore, we can assume that the temperature dependence of one BCF-12 PSD is a good estimate of a set of PSDs if a uniform procedure of PSD manufacturing is respected [10].

Other PSDs using BCF-12 irradiated with different sources of radiation have also shown negative temperature dependencies of −0.15%/°C (50 kVp) [19], −0.09%/°C (Co-60) [20], −0.225%/°C (6 MV, 15 MV) [17], −0.263%/°C (50–150 kVp) [21], −0.25%/°C (6 MV, 10 MV, 15 MV, Co-60) [16], −0.18%/°C (6 MV) [22], and −0.18%/°C (6 MV FFF) [15]. The mean value of all these works is around −0.2%/°C, which coincides with the value obtained in the present work considering the associated uncertainty. An alternative to PSD, high-Z inorganic detectors, show a much higher temperature dependence of −1.4%/°C [35].

Manual correction for the difference between calibration and patient temperatures for IVD is straightforward using the data from Figure 4 with which a correction factor can be determined and applied to the calibration coefficient of the detector. Once the corrected calibration factor is applied, if the patient of the in vivo measurement has a slightly different temperature from normothermia, the low temperature dependence of BCF-12 PSD assures a negligible modification of the correction factor. For example, a variation of 1 °C in the patient temperature would result in a variation of ±0.2%, which is perfectly assumable. The strength of the method used for temperature dependence measurements in this study was that two temperature sensors were used, one above the PSD and one below, to better characterize the temperature of the PSD.

For in vivo gynecologic brachytherapy applications, a single correction factor or calibration for normothermia of ~37 °C should be sufficient [36]. Rectal temperature can be considered similar to that of the vagina. The rectal temperature of a healthy adult woman has a standard deviation (SD) of 0.36 °C. Considering two SDs, we could assume that the vaginal temperature of any patient is ~37 °C with a 0.7 °C uncertainty (*k* = 2). Based on our results, for PSD temperature dependence, this uncertainty would translate into an uncertainty of <0.2% in signal for BCF-12. This value is considerably smaller than other uncertainties that might be encountered in IVD (for example, uncertainty in the detector location due to the difficulty of reproducibly, placement, or calibration factor uncertainties).

One limitation of the present study is the high experimental positioning uncertainty of the source in the catheter at short distances between the detector probe and the Ir-192 source in the tests of energy and angular dependence. The source could be positioned at an accuracy of 1.0 mm by the afterloader. However, we have to assume another ±1 mm uncertainty in catheter connection to the transfer tube. In the case of the energy dependence measurements, at short distances this uncertainty translates into large absorbed dose rate uncertainties, as can be observed in Figure 2. 

## 5. Conclusions

The present PSD was evaluated in various set-ups to characterize its performance. The results show promising results as compared with the published data, and the dependencies to all parameters investigated were reported. This study demonstrates that when used with robust calibration and corrected for the energy, angular, and temperature dependence factors, the PSD constitutes an excellent dosimeter compatible with IVD in brachytherapy.

## Figures and Tables

**Table 1:** This table is a summary of the Monte Carlo methods used.

Topic	Item	Data
Software	Code, version/ release date	PENELOPE/PenEasy [23] 25 March 2020
Hardware	CPU model CPU time	Intel Core i7-7700 8.7 × 10^6^ s for $D_{pol}^{full\;scatter}$ 4.3 × 10^6^ s for $D_{pol}^{PMMA\;phantom}$ 6.6 × 10^6^ s for $D_{w}^{full\;scatter}$ 3.8 × 10^6^ s for $D_{w}^{PMMA\;phantom}$ 5.1 × 10^6^ s for $D_{air}^{full\;scatter}$ 2.7 × 10^7^ s for $D_{air}^{PMMA\;phantom}$
Geometry	Geometry 1 Geometry 2 PSD Farmer chamber	10 cm × 10 cm cylindrical PMMA phantom Water sphere of radius 40 cm [25] 0.5 mm diameter, 2 mm length Data provided by the manufacturer [27]
Materials	Air composition ^1^ Air density ^2^ Water density ^3^ Farmer chamber	75.527% N, 23.178% O, 1.283% Ar, 0.012% C 1.205 mg/cm^3^ was used for the standard temperature of 20 °C and an atmospheric pressure of 101.325 kPa Degassed liquid water ρ = 0.998 g/cm^3^ at 20 °C Data provided by the manufacturer [27]
Source	Ir-192 source Ir-192 spectrum	Model used and parameter values [24] National Nuclear Data Center (NNDC) [29]
Physics and transport	Electrons’ cut-off energy Photons’ cut-off energy Optimization ^4^ Variance reduction technique ^5^	1 keV in the detector volume and 10 keV in the rest of the phantom. 1 keV in all volumes 8.4 cm^3^ ellipsoid external to the detector Interaction forcing IF = 200
Scoring	Scored quantities Number of histories ^6^	Energy deposited per history in the detector (eV/history) ~3 × 10^11^ s histories

^1^ It differs slightly from the recommended in report ICRU 90 [28]. ^2^ Recommended in supplement 2 for the 2004 update of the AAPM Task Group No. 43 Report [6]. ^3^ There is no difference in density compared to the corresponding 22 °C value [7]. ^4^ To optimize the simulations, an additional 8.4 cm^3^ ellipsoid external to the detector was generated. The size of the same was selected so that the secondary electrons generated outside the volume did not reach the detector. Only particles in that volume were used for the transport of radiation in the detector. ^5^ It allows to us reduce the calculation time considerably. This technique generates forced interactions along the particle path. To compensate for this excess probability of interaction, the average free path is proportionally reduced, and an appropriate statistical weight is assigned to any secondary particle or energy loss resulting from those interactions. ^6^ The number of histories was the minimum number necessary to achieve an uncertainty under 1%.

**Table 2:** Estimated uncertainties (*k* = 1) of the Monte Carlo PENELOPE/PenEasy simulations.

	Relative Propagated Uncertainty	
Uncertainty Component	Type A [%]	Type B [%]
${\left(s_{w,air}\cdot p\cdot W_{air}\right)}_{Co-60}$ ^1^	-	0.8
Clinic Monte Carlo ^2^	-	1.6
Phantom composition, density ^3^	-	0.6
Materials’ cross-sections ^4^	-	0.1
Total combined uncertainty	1.9%	

^1^ From IAEA report TRS-398 [26]. ^2^ From Granero D et al. 2011, Medical Physics [31]. ^3^ From Reed J-L et al. 2014 Brachytherapy [32]. ^4^ From Andreo P et al. 2012 Phys Med Biol [33].

**Table 3:** Estimated uncertainties (*k* = 1) of the Farmer chamber ionization measurements.

	Relative Propagated Uncertainty	
Uncertainty Component	Type A [%]	Type B [%]
$N_{D,w,Co-60}$ calibration factor ^1^	-	0.6
Electrometer calibration ^1^ Clinic Farmer reproducibility	- 0.5	0.1 -
$k_{s}\cdot k_{pol}\cdot k_{P,T}$ Source positioning inside needles	- -	0.2 1.6
Mechanical drilling of needle holes	-	1.0
Total combined uncertainty	2.1%	

^1^ Obtained from PTW calibration certificates.

**Table 4:** Monte Carlo simulation ratios in both geometries and their experimental validation with coverage factor *k* = 1.

Ratio	Monte Carlo Result ^1^	Experimental Validation
$D_{pol}^{\;\;\;\;\;full\;scatter}/D_{pol}^{\;\;\;\;\;\;PMMA\;phantom}$	1.075 ± 2.4%	1.084 ± 2.1% ^2^
$D_{w}^{\;\;\;full\;scatter}/D_{w}^{\;\;\;\;PMMA\;phantom}$	1.096 ± 2.4% ^3^	-
$D_{air}^{\;\;\;\;\;\;full\;scatter}/D_{air}^{\;\;\;\;\;\;PMMA\;phantom}$	1.086 ± 2.4% ^4^	1.106 ± 2.9% ^5^

^1^ The uncertainty was obtained from Table 2 components excluding ${\left(s_{w,air}\cdot p\cdot W_{air}\right)}_{Co-60}.$ For the three ratios, the uncertainties corresponding to the phantom composition, density, and materials’ cross-sections are considered the same. ^2^ Only the Type A uncertainty component was considered based on 10 consecutive acquisitions. ^3^ This result was obtained in the same conditions as the polystyrene ratio except for the detector material: water instead polystyrene. ^4^ This Monte Carlo ratio was obtained from the mean absorbed dose in the sensitive volume of air of the Farmer model in both phantoms. ^5^ This experimental ratio was obtained from a series of ten measurements in the water scanning phantom and in the PMMA phantom.

## Data Availability

The data presented in this study are available upon request from the corresponding author.

## References

[1] Fonseca G.P., Johansen J.G., Smith R.L., Beaulieu L., Beddar S., Kertzscher G., Verhaegen F., Tanderup K. (2020). In vivo dosimetry in brachytherapy: Requirements and future directions for research, development, and clinical practice. Phys. Imaging Radiat. Oncol..

[2] Herreros A., Pérez-Calatayud J., Ballester F., Barrera-Gómez J., Abellana R., Melo J., Moutinho L., Tagliaferri L., Rovirosa Á. (2022). In Vivo Verification of Treatment Source Dwell Times in Brachytherapy of Postoperative Endometrial Carcinoma: A Feasibility Study. J. Pers. Med..

[3] Belley M.D., Craciunescu O., Chang Z., Langloss B.W., Stanton I.N., Yoshizumi T.T., Therien M.J., Chino J.P. (2018). Real-time dose-rate monitoring with gynecologic brachytherapy: Results of an initial clinical trial. Brachytherapy.

[4] Rosales H.M.L., Duguay-Drouin P., Archambault L., Beddar S., Beaulieu L. (2019). Optimization of a multipoint plastic scintillator dosimeter for high dose rate brachytherapy. Med. Phys..

[5] Perez-Calatayud J., Ballester F., Tedgren Å.C., DeWerd L.A., Papagiannis P., Rivard M.J., Siebert F.-A., Vijande J. (2022). GEC-ESTRO ACROP recommendations on calibration and traceability of HE HDR-PDR photon-emitting brachytherapy sources at the hospital level. Radiother. Oncol..

[6] Rivard M.J., Ballester F., Butler W.M., DeWerd L.A., Ibbott G.S., Meigooni A.S., Melhus C.S., Mitch M.G., Nath R., Papagiannis P. (2017). Supplement 2 for the 2004 update of the AAPM Task Group No. 43 Report: Joint recommendations by the AAPM and GEC-ESTRO: Joint. Med. Phys..

[7] Perez-Calatayud J., Ballester F., Das R.K., DeWerd L.A., Ibbott G.S., Meigooni A.S., Ouhib Z., Rivard M.J., Sloboda R.S., Williamson J.F. (2012). Dose calculation for photon-emitting brachytherapy sources with average energy higher than 50 keV: Report of the AAPM and ESTRO. Med. Phys..

[8] Beaulieu L., Beddar S. (2016). Review of plastic and liquid scintillation dosimetry for photon, electron, and proton therapy. Phys. Med. Biol..

[9] Beddar S., Beaulieu L. (2016). Scintillation Dosimetry.

[10] Alharbi M., Gillespie S., Woulfe P., Mccavana P., O’Keeffe S., Foley M. (2019). Dosimetric Characterization of an Inorganic Optical Fiber Sensor for External Beam Radiation Therapy. IEEE Sensors J..

[11] Cusumano D., Placidi L., D’Agostino E., Boldrini L., Menna S., Valentini V., De Spirito M., Azario L. (2020). Characterization of an inorganic scintillator for small-field dosimetry in MR-guided radiotherapy. J. Appl. Clin. Med. Phys..

[12] Ruiz-Arrebola S., Fabregat-Borrás R., Rodríguez E., Fernández-Montes M., Pérez-Macho M., Ferri M., García A., Cardenal J., Pacheco M.T., Anchuelo J. (2020). Characterization of microMOSFET detectors for in vivo dosimetry in high-dose-rate brachytherapy with ^192^Ir. Med. Phys..

[13] Fröhlich G., Kovács K.D., Major T., Polgár C. (2019). In vivo dosimetry of the rectum in image-guided adaptive interstitial-intracavitary brachytherapy of cervix cancer—A feasibility study. Rep. Pract. Oncol. Radiother..

[14] Ferrer C., Huertas C., García D., Sáez M. (2023). Dosimetric characterization of a novel commercial plastic scintillation detector with an MR-Linac. Med. Phys..

[15] Jacqmin D.J., Miller J.R., Barraclough B.A., Labby Z.E. (2022). Commissioning an Exradin W2 plastic scintillation detector for clinical use in small radiation fields. J. Appl. Clin. Med. Phys..

[16] Dimitriadis A., Patallo I.S., Billas I., Duane S., Nisbet A., Clark C. (2017). Characterisation of a plastic scintillation detector to be used in a multicentre stereotactic radiosurgery dosimetry audit. Radiat. Phys. Chem..

[17] Carrasco P., Jornet N., Jordi O., Lizondo M., Latorre-Musoll A., Eudaldo T., Ruiz A., Ribas M. (2015). Characterization of the Exradin W1 scintillator for use in radiotherapy. Med. Phys..

[18] Rosales H.M.L., Archambault L., Beddar S., Beaulieu L. (2020). Dosimetric performance of a multipoint plastic scintillator dosimeter as a tool for real-time source tracking in high dose rate ^192^Ir brachytherapy. Med. Phys..

[19] Buranurak S., Andersen C.E., Beierholm A.R., Lindvold L.R. (2013). Temperature variations as a source of uncertainty in medical fiber-coupled organic plastic scintillator dosimetry. Radiat. Meas..

[20] Wootton L., Beddar S. (2013). Temperature dependence of BCF plastic scintillation detectors. Phys. Med. Biol..

[21] Lee B., Shin S.H., Jang K.W., Yoo W.J. (2015). Effects of temperature and X-rays on plastic scintillating fiber and infrared optical fiber. Sensors.

[22] Galavis P.E., Hu L., Holmes S., Das I.J. (2019). Characterization of the plastic scintillation detector Exradin W2 for small field dosimetry. Med. Phys..

[23] Sempau J., Badal A., Brualla L. (2011). A PENELOPE-based system for the automated Monte Carlo simulation of clinacs and voxelized geometries—Application to far-from-axis fields. Med. Phys..

[24] Ballester F., Tedgren Å.C., Granero D., Haworth A., Mourtada F., Fonseca G.P., Zourari K., Papagiannis P., Rivard M.J., Siebert F.-A. (2015). A generic high-dose rate ^192^Ir brachytherapy source for evaluation of model-based dose calculations beyond the TG-43 formalism. Med. Phys..

[25] Pérez-Calatayud J., Granero D., Ballester F. (2004). Phantom size in brachytherapy source dosimetric studies. Med. Phys..

[26] International Atomic Energy Agency (2024). Absorbed Dose Determination in External Beam Radiotherapy, Technical Reports Series No. 398 (Rev. 1).

[27] Gomà C., Sterpin E. (2019). Monte Carlo calculation of beam quality correction factors in proton beams using PENH. Phys. Med. Biol..

[28] ICRU (2016). Report 90. J. Int. Comm. Radiat. Units Meas..

[29] NUDAT 2.6 (2015). National Nuclear Data Center, Brookhaven National Laboratory. http://www.nndc.bnl.gov/nudat2/.

[30] JCGM 2008:100. Evaluation of measurement data—Guide to the expression of uncertainty in measurement. Int Organ Stand Geneva ISBN. 2008; (September): 134. http://www.bipm.org/en/publications/guides/gum.html.

[31] Granero D., Vijande J., Ballester F., Rivard M.J. (2011). Dosimetry revisited for the HDR ^192^Ir brachytherapy source model. Med. Phys..

[32] Reed J.L., Rivard M.J., Micka J.A., Culberson W.S., DeWerd L.A. (2014). Experimental and Monte Carlo dosimetric characterization of a 1 cm 103Pd brachytherapy source. Brachytherapy.

[33] Andreo P., Burns D.T., Salvat F. (2012). On the uncertainties of photon mass energy-absorption coefficients and their ratios for radiation dosimetry. Phys. Med. Biol..

[34] Gonod M., Suarez M.A., Avila C.C., Karakhanyan V., Eustache C., Crouzilles J., Laskri S., Vinchant J.-F., Aubignac L., Grosjean T. (2022). Characterization of a miniaturized scintillator detector for time-resolved treatment monitoring in HDR-brachytherapy. Phys. Med. Biol..

[35] Jørgensen E.B., Johansen J.G., Overgaard J., Piché-Meunier D., Tho D., Rosales H.M.L., Tanderup K., Beaulieu L., Kertzscher G., Beddar S. (2021). A high-Z inorganic scintillator–based detector for time-resolved in vivo dosimetry during brachytherapy. Med. Phys..

[36] Geneva I.I., Cuzzo B., Fazili T., Javaid W. (2019). Normal body temperature: A systematic review. Open Forum Infect. Dis..

